# A Comprehensive Review of Novel FDA-Approved Psychiatric Medications (2018-2022)

**DOI:** 10.7759/cureus.56561

**Published:** 2024-03-20

**Authors:** Shannon Giliberto, Rhea Shishodia, Meredith Nastruz, Chamandeep Brar, Sadeepa Bulathsinhala, Jonathan Terry, Sudhakar Pemminati, Sudhakar K Shenoy

**Affiliations:** 1 Department of Biomedical Education, California Health Sciences University College of Osteopathic Medicine, Clovis, USA; 2 Department of Biomedical Education, St. George's University School of Medicine, True Blue, GRD; 3 Department of Specialty Medicine, California Health Sciences University College of Osteopathic Medicine, Clovis, USA; 4 Department of Psychiatry, Clarity Clinic, Chicago, USA

**Keywords:** bremelanotide, brexanolone, lofexidine, pitolisant, solriamfetol

## Abstract

Mental health disorders are among the top leading causes of disease burden worldwide and many patients have high levels of treatment resistance. Even though medications offer improvement to some patients, antidepressants are only effective in about half of those treated, and schizophrenia is treatment-refractory in about one-third of patients. One way to combat this disparity is to improve medication development and discovery for psychiatric disorders through evidence-based research. Recently, most psychiatric medications approved by the United States Food and Drug Administration (FDA) are for increased tolerability or extended release. Because of the slow, incremental progress, there is a pressing need to explore novel medications with new indications or mechanisms of action to treat the expanding population with mental disorders, especially in those who are fully or partially recalcitrant to first-line medication options. This review aims to present the newest FDA medications with new indications, establish the clinical need for each, and discuss future directions in drug development. We searched and reviewed novel psychiatric medications approved by the FDA from 2018 to 2022. We then analyzed each medication in the United States Clinical Trials Registry and gathered updated results for efficacy and safety information. We also searched PubMed/MEDLINE (Medical Literature Analysis and Retrieval System Online), Scopus, Web of Science, Elsevier, and Google Scholar to understand how these new indications met current clinical needs. Finally, we inquired about related technological implications that will lead the field of psychopharmacology now and in the years to come. We found 12 novel psychiatric medications approved by the FDA from 2018 to 2022, representing a very small percentage of the total FDA approvals during that period. These psychiatric medications with novel mechanisms or improved efficacy and safety  are expected to provide further options for treating mental health disorders; promising results will lead to new patterns of research.

## Introduction and background

Psychiatric medication research and development often lags behind other pharmaceutical developments [1]. Yet, mental health disorders remain among the top 10 leading causes of disease burden worldwide and many patients have high levels of treatment resistance [2,3]. Of all mental health disorders, depression and anxiety disorders are the most common causes of global disease burden. Schizophrenia and bipolar disorder are less prevalent in the global population, but disability related to psychosis has a 10-fold higher cost than general patient care [2,4]. Even though medications offer improvement to some patients, antidepressants are only effective in about half of those treated, and schizophrenia is treatment-refractory in about one-third of patients [1,4]. Mental disorders also increase the risk of adverse outcomes up to suicide. Premature mortality can also occur from infectious diseases, neoplasms, diabetes, heart disease, stroke, and respiratory disease [2]. The contrast between dire need and the paucity of research is striking.

As of December 2011, a publication by Pharmaceutical Research and Manufacturers of America (PhRMA) reported that only 240 drugs were in the pipeline for mental health, in contrast to more than 3000 for cancer and 750 for infectious disease [5]. This is disproportionate to global disability-adjusted life years (DALYs) across psychiatric disorders versus cancer. In the year 2019, across all age groups, depressive disorders were the 13th leading cause of elevated DALYs and anxiety disorders were 24th. In contrast, lung cancer is the only cancer in the top 25 causes of disability, ranking at number 17. The percentage of change in DALYs for depressive and anxiety disorders has elevated from 1990 to 2019 by 61.1% and 53.7%, respectively. Lung cancer DALYs have also elevated by 69.1%. In addition, in the age group of 10-24, depressive disorders are the fourth leading cause of DALYs right behind “self-harm.” They are also high in the age group of 25-45, resulting as the sixth leading cause of DALYs. Despite greater numbers in DALYs, new psychiatric drug development is much less than drug development for cancer [6]. Medication development is arguably one of the most significant factors limiting the potential to treat mental disorders more effectively. One way to combat this disparity is to emphasize improving medication development and discovery for psychiatric disorders through evidence-based research; however, drug development and approval are complex. Most new applications come from existing drugs with new indications including formal recognition of off-label uses [3]. 

We searched and reviewed novel psychiatric medications approved by the FDA in the 2018-2022 timeframe. We then analyzed each medication in the United States Clinical Trials Registry and gathered updated results for efficacy and safety information. We also searched PubMed to understand how these new indications met current clinical needs.  Tables for the FDA-approved medications are presented, including approval dates, manufacturer, active ingredient, target, mechanism of action, indication, and adverse events. Finally, through  PubMed/MEDLINE (Medical Literature Analysis and Retrieval System Online), Scopus, Web of Science, Elsevier, and Google Scholar, we inquired about related technological implications that will lead the field of psychopharmacology now and in the years to come. 

## Review

**Figure 1:**
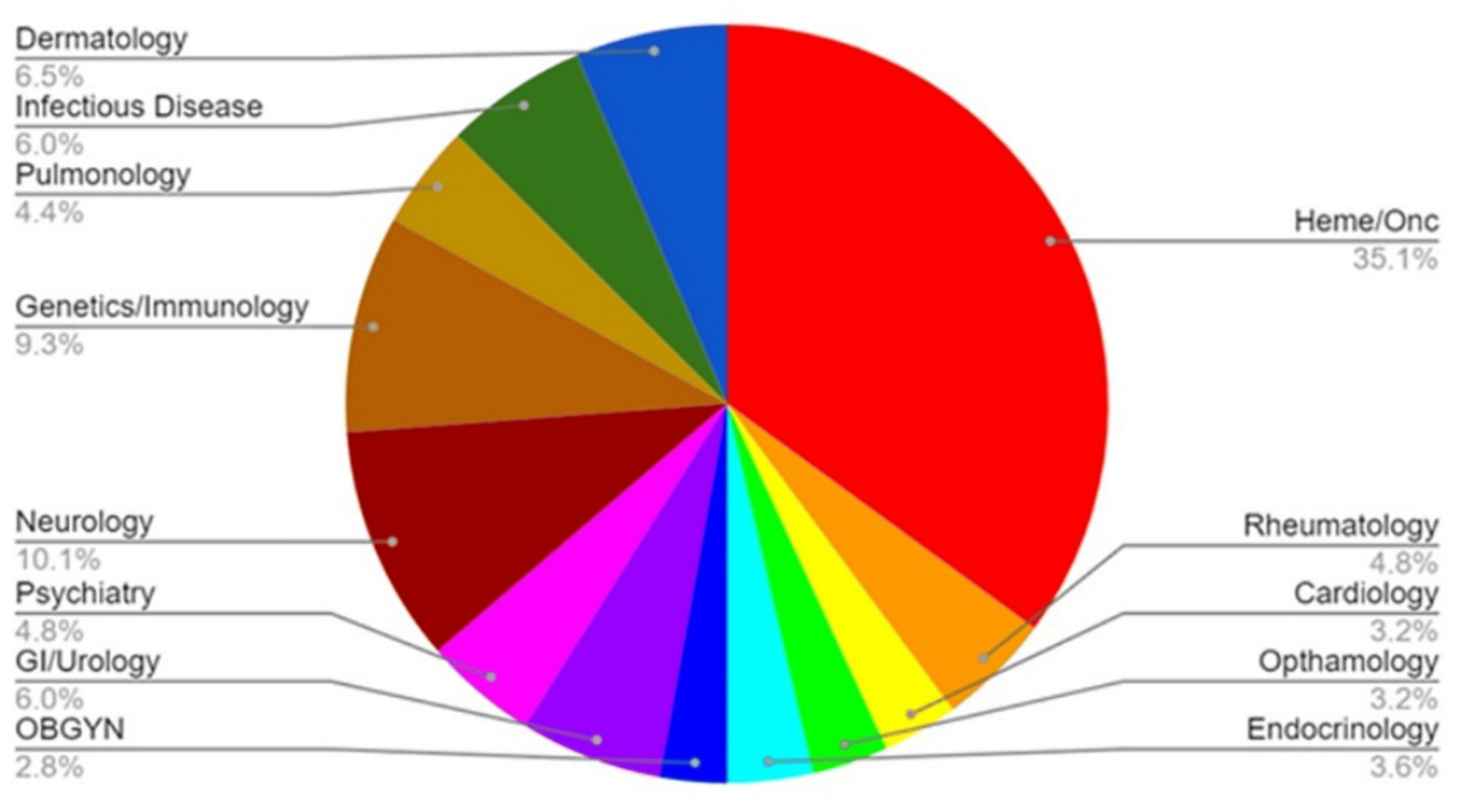
Pharmaceutical agents approved by FDA in 2018-2022, segregated by specialty OBGYN: obstetrics and gynecology Image credit: Shannon Giliberto

We found 12 novel psychiatric medications out of 248 total pharmaceutical agents approved by the FDA from 2018 to 2022, representing 4.8% of total FDA approvals during that period (Figure 1). In 2018, lofexidine was approved for the treatment of opioid withdrawal. In 2019, brexanolone was approved for postpartum depression (PPD), lumateperone for schizophrenia, bremelanotide (BMT) for hypoactive sexual desire disorder (HSDD), and lemborexant for insomnia. Solriamfetol and pitolisant were approved to treat excessive daytime sleepiness (EDS) for adults with narcolepsy in 2019 as well.

In 2021, serdexmethylphenidate (SDX)/dexmethylphenidate (d-MPH) and viloxazine were both approved by FDA for  attention-deficit/hyperactivity disorder (ADHD), and olanzapine/samidorphan (OLZ/SAM) was approved to treat schizophrenia and aspects of bipolar 1 disorder. The latest FDA approvals of 2022 included daridorexant for insomnia and dextromethorphan/bupropion (DXM/BUP) for major depressive disorders (MDDs) [7]. Of note, medications that reduce the side effects of neurodegenerative disorders were excluded. Also, only four out of the 12 medications used a different mechanism to treat a disorder rather than being considered a new indication (first-in-class). These include lofexidine, BMT, brexanolone, and pitolisant. Two of these medications are the first drugs to be approved by FDA for a condition that had few to no medication developments in the past, including BMT for HSDD and brexanolone for PPD. Clinical trials suggested favorable results in Phase III trials that led to approval, along with current success in Phase IV trials.

In the year 2018

Lofexidine For Opioid Withdrawal

In May 2018, lofexidine became the first FDA-approved non-opioid treatment to mitigate opioid withdrawal symptoms in adults [8,9]. Lofexidine is a central alpha-2 adrenergic receptor agonist. Previously, lofexidine had been indicated for the treatment of hypertension along with clonidine [8,10,11]. The FDA considers lofexidine a first-in-class medication as it is the first alpha-2 agonist to be approved for opioid withdrawal. Lofexidine offers a non-opioid alternative to the treatment of withdrawal symptoms without a significant impact on blood pressure relative to clonidine [12].

In a study by Yu et al., a Phase-III double-blind trial was conducted across three sites with 68 opioid-dependent subjects according to the Diagnostic and Statistical Manual of Mental Disorders, fourth edition (DSM-IV) criteria [13]. Thirty-five of those subjects were randomized to lofexidine and 33 were given the placebo. The study consisted of three phases and was split into 11 days. The primary outcome measure used was the Modified Himmelsbach Opiate Withdrawal Scale (MHOWS), where lower scores indicate fewer withdrawal symptoms. By day 5 of the study, the MHOWS scores showed that the group randomized to lofexidine had a much lower score than those taking the placebo. The results were significant in demonstrating the effectiveness of lofexidine versus placebo; therefore, the study was terminated early by the Data and Safety Monitoring Board due to the belief that continuing with participants on the placebo would not be ethical given the results thus far. Adverse effects found in the study included  hypotension (18%), dizziness (39%), asthenia (61%), and insomnia (79%) [13]. 

In a second study by Gorodetzky et al., an eight-day double-blind trial took place with 264 participants (134 on lofexidine and 130 on placebo). Treatment with lofexidine or the placebo was given four times daily during days 1-5. On days 6 and 7, a placebo was given to all the participants, and on day 8, no medication was administered. The two primary outcome measurements were the Short Opiate Withdrawal Scale (SOWS)-Gossop score on the third day of treatment and time-to-dropout. The results of the study showed that the SOWS scores for the third day of treatment were lower in the lofexidine group when compared to the placebo group, and when comparing the two groups, fewer participants of the lofexidine group dropped out of the study than those in the placebo group. Adverse effects of this study included hypotension (25.4%), bradycardia (9.7%), and dizziness (22.4%) [14]. 

In a third study, Fishman et al. conducted a double-blind study that had 602 participants: 151 received the placebo, 229 received the 2.16 mg/day lofexidine, and 222 received the 2.88 mg/day lofexidine over seven days. The primary outcome was measured through the SOWS-Gossop scale. The results indicate that there is a statistically significant difference in the mean SOWS-Gossop score when comparing both lofexidine groups to the placebo. The adverse effects in this study include orthostatic hypotension (29.3% in 2.16 mg group, 42.3% in 2.88 mg group), insomnia (51.1% in 2.16 mg group, 55.4% in 2.88 mg group), bradycardia (23.6 % in 2.16 mg group, 31.5% in 2.88 mg group), and dizziness (19.2% in 2.16 mg group, 23.0% in 2.88 mg group) [15]. 

Overall, these studies consistently show that lofexidine is efficacious for managing opioid withdrawal symptoms as it is a first-in-class drug, and it puts forth another tool to use when working with patients to find a treatment plan that best fits them [8,12,16]. The findings are summarized in Table 1. 

**Table 1:** Medications approved by FDA in 2018

Approval date	Brand name	Active ingredient	Mechanism of action	Indication	Adverse effects
May 16, 2018	Lucemyra	Lofexidine hydrochloride	Central alpha-2 agonist	Non-opioid treatment for the management of opioid withdrawal symptoms	Hypotension (30%), Dizziness (21%), Insomnia (53%), Bradycardia (27%)

In the year 2019

BMT For HSDD

In June 2019, BMT was approved to treat HSDD in premenopausal women [17]. BMT which is a melanocortin receptor (MCR) agonist for five subtypes: MC1R, MC4R, MC3R, MC5R, and MC2R. When given at therapeutic dose levels MC1R and MC4R hold the greatest  affinity. There are several areas in the central nervous system that express MC4R  [18]. BMT improves HSDD by stimulation of MCR which leads to increased dopamine release, thereby enhancing sexual desire and response. When BMT was approved by the FDA, it was the second drug indicated for use in HSDD. The first drug was flibanserin.  A major difference  between the two treatments was that BMT is used subcutaneously through an auto-injector as needed, and flibanserin is taken in an oral tablet form daily. Another major difference is the mechanism of action of flibanserin. It has agonist activity at 5-HT1A receptors and antagonist activity at 5-HT2A receptors [18,19,20]. 

In a study by Clayton et. al., a double-blind Phase-IIB trial was conducted to analyze the safety and efficacy of BMT and to find the doses that would be suited to be tested in the Phase-III trials. The patient population consisted of non-pregnant women of at least 21 years of age who had been diagnosed with HSDD, female sexual arousal disorder (FSAD), or both. The participants were randomized into four categories: placebo, BMT 0.75 mg dose, BMT 1.25 mg dose, and BMT 1.75 mg dose. The primary measure outcomes were the patients' change from baseline to the end of the study in the number of satisfying sexual events (SSE) through the Female Sexual Encounter Profile-Revised (FSEP-R) questionnaire. Other measure outcomes include a change from baseline to end of study in total Female Sexual Function Index (FSFI) score and Female Sexual Distress Scale-Desire/Arousal/Orgasm (FSDS-DAO) score. An increase in scores from baseline in FSFI indicates improvement. A decrease in scores from FSDS-DAO indicates improvement. The results indicated that the 1.25 and 1.75 mg dose groups had improvement in the SSEs per month and also had improvements in the FSFI and FSDS-DAO scores. The 0.75 mg dose did not produce improvements. Thus, the 1.25 mg and 1.75 mg were the effective doses in this trial. Side effects in this study included nausea, headache, and flushing [20]. 

Two identical Phase-III double-blind trials were conducted. In these two studies, a 1.75 mg dose of BMT or placebo was administered subcutaneously via an auto-injector to women in the age group of 18-52  years, who had been diagnosed with HSDD [21]. The BMT or placebo was to be taken on an as-needed basis with limiting one dose in 24 hours over 24 weeks. The primary measure outcomes for both studies was a change from baseline to end of study in total FSFI score and FSDS-DAO score. Studies 1 and 2 began in 2015, and the results of both studies indicated that patients who took the BMT had increases in sexual desire indicated by the FSFI score when compared to the placebo. In addition, there were also decreases in distress stemming from low sexual desire as indicated by the FSDS-DAO score when compared to the placebo. The adverse effects of BMT indicated in these studies are nausea, flushing, and headaches [21-24]. 

These studies indicate that BMT is effective in treating HSDD in pre-menopausal women. Limitations include untested safety and efficacy in post-menopausal women and women with psychiatric disorders who require medications [21]. 

Brexanolone For PPD

In March 2019, brexanolone became the first FDA-approved treatment specifically for PPD. Brexanolone is solely given to patients at healthcare facilities with restricted distribution programs allowing monitoring of IV administration of the medication and ensuring adherence with a Risk Evaluation and Mitigation Strategy (REMS) [25,26]. Brexanolone is a  neuroactive steroid GABA-A receptor-positive modulator [26]. It has the same structure as endogenous allopregnanolone, which has been associated with antidepressant and anti-anxiety effects. Brexanolone stimulates more phasic and tonic inhibition at the synaptic and extrasynaptic GABA-A receptors. The dysregulated GABAergic neural activity has been associated with PPD, and through modulation by brexanolone, it may improve symptoms of depression [27,28]. 

In a double-blind randomized study conducted by Kanes et al., female participants less than six months postpartum had severe depression according to the Hamilton Rating Scale for Depression (HAM-D) with a total score of greater or equal to 26. A lower HAM-D score indicates less severe depressive episodes. Participants were given either a continuous intravenous dose of brexanolone or a placebo for 60 hours followed by a final HAM-D total score at 60 hours to compare each patient’s baseline. Twenty-one women were enrolled in the study, 10 of whom were assigned to brexanolone and 11 to the placebo. At the end of 60 hours, the HAM-D score dropped 20.97 points from the baseline for the brexanolone group, and the HAM-D score dropped 8.75 points from the baseline for the placebo group. The adverse effects found in this study include dizziness, sinus tachycardia, and somnolence [29]. 

Two studies by Melter-Brody et al. were conducted in 2017, and the primary measure of outcome for both studies was the change from baseline in the 17-item HAM-D. In Study 1, there were a total of 138 participants, and it demonstrated that the group assigned to brexanolone 60 micrograms per kilogram per hour (BRX60) had the greatest mean reduction in HAM-D total score when compared to the BRX90 group and the placebo group. Study 2 had 108 participants, and it demonstrated that the BRX90 group had a greater mean reduction in HAM-D total score when compared to the placebo. Some adverse effects in these studies included headache, dizziness, and somnolence [30]. 

In effect, these studies have shown that brexanolone is effective in reducing PPD symptoms; however, there may be a barrier to access due to the cost of treatment related to requisite in-patient hospitalization for patient monitoring. Overall, brexanolone is a first-in-class drug, and it is a starting point that will allow for more cost-effective treatments to come forward [31,32]. 

*Solriamfetol For EDS And Narcolepsy* 

Solriamfetol is a central nervous system stimulant that was approved by the FDA in March 2019 for its use of the active ingredient solriamfetol to manage EDS associated with narcolepsy or obstructive sleep apnea (OSA) in adults.  Solriamfetol  works by increasing the levels of dopamine and norepinephrine by inhibiting presynaptic reuptake of norepinephrine and dopamine in the brain, which can help promote wakefulness and reduce daytime sleepiness. 

The FDA approval of solriamfetol was based on the results of two randomized, double-blind, placebo-controlled Phase-III clinical trials involving over 1,000 participants with narcolepsy or OSA who experienced EDS. Participants were randomly assigned to receive either solriamfetol or placebo once daily in the morning for 12 weeks. The primary endpoint of these studies was the change in the Epworth Sleepiness Scale (ESS) score, which is a validated measure of daytime sleepiness, from baseline to week 12. Secondary endpoints included measures of wakefulness, sleep quality, and overall functioning. Study 1 showed that solriamfetol 75 mg and 150 mg resulted in a mean change in ESS score of -3.8 and -5.4 points, respectively, compared to -1.6 points for placebo. The second study demonstrated that solriamfetol  37.5 mg (the starting dose for OSA) and  75 mg (the recommended starting dose for narcolepsy) resulted in a mean change in ESS score of -5.1 and -5.0 points, respectively, compared to -3.3 points for placebo. The results of these studies extrapolated that solriamfetol significantly (p<0.05) improved EDS compared to placebo, as measured by the change in ESS score. Participants who received solriamfetol had a greater reduction in ESS score and a greater increase in wakefulness and overall functioning compared to those who received placebo. Solriamfetol was also generally well-tolerated, with the  most common adverse effects  being headache, nausea, anxiety, insomnia, irritability, dizziness, and palpitations [33]. 

Overall, the clinical trials suggest that solriamfetol is an effective and safe treatment option for adults with narcolepsy or OSA who experience EDS. The approval of solriamfetol represents an important new treatment option for people with narcolepsy or OSA who experience EDS, as it provides a  non-amphetamine-based option for improving wakefulness and reducing daytime sleepiness. Non-amphetamine drugs have several advantages over amphetamines, including a lower risk of dependency and abuse, fewer and less severe side effects, a longer duration of action, less impact on sleep, less stringent regulation, less likelihood of causing tolerance, and a broader range of therapeutic applications. These privileges make non-amphetamine drugs a valuable alternative in many treatment scenarios. 

Pitolisant For Narcolepsy 

Pitolisant was approved by the European Union in March 2016 for narcolepsy with or without cataplexy, and for the use of EDS by the FDA in August 2019 [34]. Pitolisant is a  histamine type 3 (H3) antagonist and inverse agonist causing an endogenous histamine increase in the cerebral cortex, hypothalamus, hippocampus, and basal ganglia by inhibiting histamine reuptake [34].  Pitolisant works as effectively as modafinil for narcolepsy but is more effective against cataplexy, symptoms such as loss of muscle control or muscle weakness.  Pitolisant was determined a first-in-class medication due to its high potential to improve symptoms by the FDA’s Center for Drug Evaluation and Research (CDER) as of 2019 [32]. This suggests that, though pitolisant is new, it could be equally or more effective at treating patients per FDA recommendations and it used a different mechanism of action than existing therapies [32]. Pitolisant is also now the only anti-narcoleptic not on the controlled substance list in the United States due to its minimal abuse risk [35].  Adverse effects of   pitolisant include  headache, insomnia, anxiety, abdominal discomfort, and QT prolongation. Pitolisant is contraindicated in  bradyarrhythmia [35,36]. 

In a double-blind placebo study conducted by  Dauvilliers et al. in 2013, across five European countries, participants included 95 adults with no psychostimulants in their system and diagnosed EDS evaluated with an ESS score of 14 or more [36].  Participants were randomized to groups of pitolisant, modafinil, or a placebo for eight weeks of treatment [36]. According to ESS scores at the end of the trial, pitolisant showed significantly better scores than the placebo (difference -3, p=0.024) and similar results to modafinil with low significance (difference=0.12, p=0.25) [36]. Of the total significant adverse events during the trial, five were from modafinil while only one was abdominal discomfort from pitolisant [36].  A second double blind-placebo study performed by  Pepin et al. in 2021 across multiple European countries included 244 obstructive sleep apnea participants with continuous positive airway pressure treatment and residual EDS [37].  These patients were randomly assigned pitolisant or a placebo, ultimately discovering that pitolisant significantly decreased the ESS score compared to placebo (-2.6, p<.001) [37].  Adverse events were reported by 47% of the participants in Pepin’s study, including headache and insomnia, though no significant adverse effects were reported [37]. Pitolisant is demonstrated through these studies to be an effective medication for EDS and narcolepsy, especially as a non-addictive and well-tolerated treatment option. 

Lumateperone For Schizophrenia and Depressive Episodes Associated with Bipolar I or II 

Lumateperone is an oral, once-daily atypical antipsychotic that was FDA-approved in late December 2019 for its use of the active ingredient lumateperone for the treatment of  schizophrenia and depressive episodes associated with bipolar I or II disorder (bipolar depression)  in adults as monotherapy and as adjunctive therapy with lithium or valproate. Lumateperone acts as an antagonist with high binding affinity at serotonin 5-HT2A receptors, as an antagonist with moderate binding affinity at postsynaptic D2 receptors, and as an inhibitor of serotonin reuptake. It also acts as a partial agonist with moderate affinity at D1 receptors (which may contribute to the indirect activation of AMPA and NMDA receptors) and at presynaptic D2 receptors with selectivity in targeting mesolimbic brain region. Lumateperone spares the nigrostriatal region, thus offering a lower risk of extrapyramidal adverse effects. These receptors are believed to play an important role in schizophrenia, bipolar disorder, depressive disorders, and other neuropsychiatric disorders [38]. FDA approval of lumateperone was acquired after the conclusion of clinical studies that evaluated its efficacy as monotherapy and adjunctive therapy with lithium or valproate for the treatment of schizophrenia and bipolar depression. 

Three randomized, double-blind, placebo-controlled, multicenter clinical trials were conducted in 450 adult patients who met the Diagnostic and Statistical Manual of Mental Disorders, Fifth Edition, (DSM-V) criteria for schizophrenia. The primary objective of all three trials was to assess the change in the Positive and Negative Syndrome Scale (PANSS) total score from baseline to the end of the treatment period: the higher the score, the greater the overall symptom severity. The results showed that lumateperone was associated with a statistically significant improvement in the PANSS total score compared to placebo as patients in the lumateperone group had a  mean reduction of 20.6 points in the PANSS total score, while patients in the placebo group had a mean reduction of 13.9 points over a total of 28 days.  Lumateperone was also associated with statistically significant improvements in other secondary endpoints, including the Clinical Global Impressions - Severity (CGI-S) scale and the Personal and Social Performance (PSP) scale. Lumateperone was generally well tolerated without clinically significant treatment-emergent motor adverse effects or changes in cardiometabolic or endocrine factors vs placebo. The most common adverse reactions in clinical trials were somnolence/sedation, dizziness, nausea,  constipation, and dry mouth [39,40]. 

As for bipolar depression, two separate clinical trials were conducted to evaluate the efficacy of lumateperone as both monotherapy and adjunctive therapy with lithium or valproate in adult patients who met DSM-V criteria for depressive episodes associated with bipolar I or bipolar II disorder. The primary efficacy measure for both trials was the change from baseline in Montgomery-Asberg Depression Rating Scale (MADRS) total score at week 6. 

As monotherapy, the efficacy of lumateperone was assessed in a six-week, randomized, double-blind, placebo-controlled, multicenter study that enrolled 381 adult patients (median age 45 years) with bipolar depression. The results indicated that lumateperone was associated with a statistically significant improvement in the MADRS total score compared to placebo as patients in the lumateperone group had a mean reduction of 16.8 points in the MADRS total score, while patients in the placebo group had a mean reduction of 13.5 points [40]. 

Additionally, the efficacy of lumateperone as adjunctive therapy with lithium or valproate, was evaluated in a six-week, randomized, double-blind, placebo-controlled, multicenter study that enrolled 529 adult patients (median age 46 years) who were experiencing a major depressive episode and were already receiving stable doses of lithium or valproate as their primary mood stabilizer. The results showed a trend for a dose-related improvement in symptoms of depression as determined by the mean reduction of 11.2 points in the MADRS total score for patients in the lumateperone group, while patients in the placebo group had a mean reduction of 6.6 points [41]. 

Lumateperone was also associated with statistically significant improvements in other secondary endpoints for both trials, including the CGI-S scale and the Sheehan Disability Scale (SDS).  Adverse events were mostly mild to moderate and similar to those seen in prior studies for schizophrenia, with no new adverse events observed. In addition, when taken with lithium, the incidence of adverse effects increases. These findings provide further evidence supporting lumateperone’s favorable safety and tolerability profile across different patient populations. 

In essence, the unique pharmacologic mechanisms of lumateperone seem to confer antipsychotic efficacy with favorable safety and tolerability. The efficacy and safety profiles of lumateperone may differ in important ways from existing treatments for patients with schizophrenia and bipolar depression.

*Lemborexant For Insomnia* 

Lemborexant is a dual orexin receptor antagonist (DORA) that was approved by FDA in December 2019 to treat adult patients with insomnia [42]. Lemborexant is a novel drug that works by directly regulating neuropeptides that control wakefulness, rather than sedating the brain. The drug's mechanism of action involves antagonism of the orexin receptors, specifically OX1R and OX2R, which are involved in the orexin neuropeptide signaling system. By blocking the binding of orexin-A and orexin-B to these receptors, lemborexant is thought to suppress the wake drive and promote sleep [43]. Two studies for lemborexant were conducted in patients with insomnia disorder who experienced difficulties with sleep onset and/or sleep maintenance. The first study was a six-month trial in adult patients randomized to receive placebo, lemborexant 5 mg, or lemborexant 10 mg once nightly. The primary endpoint was the mean change from baseline to end of treatment for log-transformed patient-reported (subjective) sleep onset latency (sSOL). The secondary endpoints were patient-reported sleep efficiency (sSEF) and wake-after-sleep onset (sWASO). Lemborexant 5 mg and 10 mg demonstrated statistically significant superiority (p < 0.05) on the primary efficacy measure, sSOL, compared to placebo, and also showed statistically significant superiority (p < 0.05) in sSEF and sWASO. The second study’s primary efficacy endpoint was the mean change in log-transformed latency to persistent sleep (LPS) from baseline to end of treatment, as measured by overnight polysomnography (PSG) monitoring. The secondary endpoints were sleep efficiency (SEF) and wake after sleep onset (WASO) measured by PSG. Lemborexant 5 mg and 10 mg demonstrated statistically significant superiority (p < 0.05) on the primary efficacy measure, LPS,  compared to placebo, and also demonstrated statistically significant improvement in SEF and WASO compared to placebo. Additionally, DORAs appear to promote both rapid eye movement (REM) and non-REM sleep, whereas GABA-A agonists, such as benzodiazepines (BZDs) and Z-drugs, promote only non-REM sleep [44]. The most common adverse effect of lemborexant is drowsiness, while serious side effects may include decreased daytime alertness, impaired ability to operate a vehicle, sleep paralysis, hallucinations, complex sleep behaviors, and worsening depression and suicidal thoughts [42,43,45].

Overall, clinical trials delineated that lemborexant has been shown to improve sleep onset and the maintenance of the sleep state in patients with insomnia. In comparison to other  insomnia medications, such as zolpidem, lemborexant has shown a  small decrease in risk of next-day impairment and reduced risk of physical dependence. However, the drug is a Schedule IV controlled substance and is contraindicated in patients with narcolepsy [46]. Due to price, the treatment option should be reserved for patients who are unable to tolerate other medications [47]. Lemborexant should also be avoided in patients with sleep apnea, chronic obstructive pulmonary disease, or depression. The findings are summarized in Table 2.

**Table 2:** Medications approved by FDA in 2019 GABA-A: gamma aminobutyric acid type A; H3: histamine type 3; 5-HT2A: 5-hydroxytryptamine type 2A; SSRI: selective serotonin reuptake inhibitor

Approval date	Brand name	Active ingredient	Mechanism of action	Indication	Adverse effects
June 21, 2019	Vyleesi	Bremelanotide	Nonselective melanocortin receptor agonist	Hypoactive Sexual Desire Disorder in Premenopausal Women	Nausea (40%), Flushing (20%), Skin hyperpigmentation (1-38%), Headache (11%)
March 19, 2019	Zulresso	Brexanolone	Allosteric modulator of GABA-A receptors	Postpartum Depression	Drowsiness (21%), Sedation (21%), Presyncope (13%)
March 20, 2019	Sunosi	Solriamfetol	Dopamine norepinephrine reuptake inhibitor	Excessive Sleepiness in Narcolepsy or Obstructive Sleep Apnea	Headache (16%), Nausea (7%), Anxiety (5%), Insomnia (5%)
August 14, 2019	Wakix	Pitolisant	H3 receptor antagonist/inverse agonist	Excessive Daytime Sleepiness in Narcolepsy, Cataplexy	Headache (18%), Insomnia (6%), Nausea (6%), Anxiety (5%)
December 20, 2019	Caplyta	Lumateperone	5-HT2A receptor antagonist SSRI D2 receptor antagonist D1 receptor partial agonist	Schizophrenia Bipolar Depression	Somnolence/Sedation (24%), Headache (14%), Nausea (8%)
December 20, 2019	Dayvigo	Lemborexant	Dual Orexin Receptor Antagonist	Insomnia	Drowsiness (8%), Fatigue (8%), nightmares (2%)

In the year 2021

SDX/d-MPH For ADHD

The central nervous system stimulant d-MPH and its prodrug SDX are available in the form of capsules [48]. This medication was approved by the FDA to treat ADHD in all patients older than five years of age as of March 2021 [48]. d-MPH has previously been used independently as a first-line ADHD treatment, as it increases dopamine and norepinephrine extracellularly and acts as an agonist at serotonin 5-HT1A receptors [48]. SDX/d-MPH has this same activity with decreased addictive properties when compared to d-MPH alone, though the combination drug is to be classified as a Class-IV controlled substance [49]. The prodrug, SDX, must be activated in the lower GI tract, suggesting a reduced risk of diversion in the body, less risk of alternative routes of administration, and overall reduced risk of abuse and substance misuse [49]. The combination SDX/d-MPH has a rapid onset of d-MPH exposure followed by a gradual decline until the next dose, leading to consistent treatment of ADHD symptoms from early morning through early evening [50]. This was proven more effective than the d-MPH extended release, which gives two peaks of symptom reduction, or the prodrug alone which led to minimal comparative reduction in overall ADHD symptoms [50,51]. According to Heal et al., SDX/d-MPH is not likely to make a large change in the psychiatric treatment of ADHD [51]. 

A double-blind, placebo-controlled study by Childress et al. reported that ADHD symptoms reduced for children aged 6-12 as indicated by the change in Swanson, Kotkin, Agler, M-Flynn, and Pelham (SKAMP) scores (14.3 with methylphenidate tablet, 25.3 with placebo, p<.0001) [52]. These participants reported adverse effects involving decreased appetite, insomnia, headache, upper abdominal pain, upper respiratory tract infection, mood swings, irritability, cough, and vomiting [52]. Concomitant use with monoamine oxidase inhibitors (MAOIs)s and phenylephrine can cause hypertensive crisis with potential outcomes including death, stroke, myocardial infarction, aortic dissection, ophthalmological complications, eclampsia, pulmonary edema, and renal failure [53, 54]. 

In a randomized control study of school-aged children with ADHD, Kollins et al. found that SDX/d-MPH improved ADHD symptoms with the Permanent Product Measure of Performance (PERMP) and SKAMP assessments with a faster onset and longer-lasting relief when compared with a placebo, roughly from hour 1 to hour 10 post-capsule administration [55]. This effect was without significant adverse events, though some experienced insomnia and decreased appetite [55].

An open-label randomized study conducted by Braeckman et al. included 23 male and female participants between the ages of 18 and 55 deemed healthy and of average weight [55]. Each subject was randomly given different SDX/d-MPH dosages (26.1/5.2 mg, SDX/d-MPH 39.2/7.8 mg, or SDX/d-MPH 52.3/10.4 mg) followed by four consecutive doses of SDX/d-MPH 52.3/10.4 mg daily for four days, with 96 hours in between each potential concentration change [55]. The single-dose studies resulted in increasing plasma concentrations of d-MPH after 13 hours with an increased initial dose (2.9 ± 1.0, 4.4 ± 1.5, and 5.7 ± 2.4 ng/mL d-MPH for 26.1/5.2 mg, 39.2/7.8 mg, or 52.3/10.4 mg SDX/d-MPH, respectively), supporting that the SMX helped to maintain the higher d-MPH concentration in the blood through the evening [55]. The majority of adverse effects in this study were minor and involved dry mouth, though there was a report of rhinorrhea [55]. Adding the prodrug SDX to d-MPH improved the effectiveness of d-MPH against ADHD symptoms while reducing the risk of addiction.

Viloxazine For ADHD

Viloxazine is a 5-HT2B receptor antagonist and 5-HT2C agonist previously used as an express-release oral medication for depression [56]. Extended-release viloxazine (viloxazine-ER) was approved as an oral medication for ADHD as of April 2021 [56]. Though it produces amphetamine-like stimulation, viloxazine is considered a non-stimulant treatment for ADHD by inhibiting both norepinephrine and serotonin reuptake [57,58]. While stimulants are still the most effective first-line or combination therapy treatment, viloxazine is best as an alternative or as a combination therapy due to side effect tolerance [56]. Adverse effects of this medication include drowsiness, decreased appetite, fatigue, nausea, vomiting, insomnia, and irritability [57]. Significant side effects include cardiovascular effects and suicidal ideation, especially if the patient has been previously diagnosed with a mental disorder and potentially seizures, which is why familial epileptic disorders should be considered before prescribing [56]. In addition, it is important to remember that viloxazine is a cytochrome P450 1A2, 2D6, 3A4, and 2B6 inhibitor [57]. If monoamine inhibitors are also used with viloxazine, or if a 14-day discontinuation period is not there between the medications, hypertensive crisis may occur [56]. In a Phase-II double-blind study with 222 participants conducted by Johnson et al, 6-12 year-old participants with ADHD were found to have significantly reduced scores on the ADHD Rating Scale (RS)-IV after a 200, 300, or 400 mg/day treatment with viloxazine-ER (p=0.031, 0.027, and 0.021, respectively). The reported adverse effects included somnolence, headache, and decreased appetite [59].

A Phase-III randomized study conducted by Nasser et al. with 477 children aged 6-11 confirmed that both a 100 and 200 mg/day treatment with viloxazine-ER is more effective for ADHD treatment than an identical placebo, confirmed with a statistically significant change from baseline (p = 0.0004 and p < 0.0001, respectively) in the ADHD-RS-5 total score at the end of the study [60]. Adverse effects ranged from mild to moderate severity in 29.6% of participants [60]. Nasser et al. conducted another Phase-III randomized double-blind study with adult subjects aged 18-65 and found that the group given flexible dosing of 200-600 mg of viloxazine had a greater reduction in their scores on the ADHD Investigator Symptom Rating Scale (AISRS), (-15.5 with viloxazine, 11.7 with placebo, p=0.004) [61]. The adverse effects reported by at least 5% of the adults in the latter study included insomnia, fatigue, nausea, decreased appetite, dry mouth, and headache, leading to 9% of subjects withdrawing from participation [62]. Viloxazine-ER is a medication newly indicated to treat ADHD that can have many side effects but offers a good non-stimulant option for patients. 

OLZ/SAM For Schizophrenia and Bipolar I Disorder

OLZ is an established atypical antipsychotic that has been widely used to treat schizophrenia and bipolar I disorder. SAM is a new drug that works by blocking the action of the mu-opioid receptor, which is involved in regulating mood and behavior. The combination of OLZ/SAM was approved by the FDA in late May 2021 to provide a more effective and well-tolerated treatment option for patients with schizophrenia and bipolar I disorder [62]. FDA approval of OLZ/SAM was obtained after the conclusion of clinical studies that evaluated its efficacy as monotherapy and adjunctive therapy with lithium or valproate for the treatment of schizophrenia and bipolar I disorder.

To evaluate the efficacy of OLZ/SAM for the treatment of schizophrenia, a four-week, randomized, double-blind, placebo- and active-controlled study was conducted which enrolled 561 adults with acute exacerbation of schizophrenia and randomized them to receive either 1:1:1 for OLZ/SAM, OLZ alone, or placebo alone for four weeks. The primary endpoint of the trial was the change from baseline to week 4 in the PANSS total score. The results showed that the combination of OLZ/SAM was non-inferior to OLZ plus placebo in reducing the PANSS total score at week 6. Moreover, the combination of OLZ/SAM resulted in a statistically significant reduction in weight gain compared to OLZ plus placebo. Patients in the OLZ/SAM group had a mean weight gain of 1.2 kg, while patients in the OLZ plus placebo group had a mean weight gain of 3.4 kg. The combination of OLZ/SAM was generally well-tolerated, with a safety profile similar to that of OLZ plus placebo. The most common adverse events reported were weight gain, somnolence, dry mouth, and suicidal ideation [63]. OLZ/SAM combination treatment was associated with significantly less weight gain and smaller increases in waist circumference than OLZ and was well tolerated. The antipsychotic efficacy of the combination treatment was similar to that of OLZ monotherapy [64].

As for bipolar I disorder, two separate clinical trials were conducted to evaluate the efficacy of OLZ as both monotherapy and adjunctive therapy with lithium or valproate. As monotherapy, the efficacy of oral OLZ in the treatment of manic or mixed episodes was demonstrated in two short-term (one three-week and one four-week) placebo-controlled studies that enrolled 124 patients with acute bipolar mania and randomized them to receive either OLZ/SAM or placebo for three weeks. The primary endpoint of the trial was the change in the Young Mania Rating Scale (YMRS) total score from baseline to week 3. The results showed that patients in the OLZ group had a significantly greater reduction in YMRS total score compared to those in the placebo group. Moreover, a significantly higher proportion of patients in the OLZ group achieved a response (≥ 50% reduction in YMRS total score) compared to the placebo group. The OLZ group also had a significantly higher proportion of patients achieving remission (YMRS total score ≤ 12) compared to the placebo group. The trial also evaluated secondary endpoints, including changes in the Clinical Global Impressions-Bipolar Version-Severity of Illness (CGI-BP-S) score and adverse events. The results showed that patients in the OLZ group had a significantly greater reduction in the CGI-BP-S score compared to the placebo group. Adverse events reported were generally mild to moderate in severity, with the most common being somnolence, dry mouth, and weight gain [63,65].

Additionally, the efficacy of oral OLZ with concomitant lithium or valproate in the treatment of manic or mixed episodes was demonstrated in a six-week double-blind, randomized, placebo-controlled trial that enrolled 344 patients with bipolar I disorder who were partially unresponsive to valproate or lithium monotherapy and randomized them to receive either OLZ plus valproate or lithium, or placebo plus valproate or lithium, for six weeks. The primary endpoint of the trial was the change in the YMRS total score from baseline to week 6. The results showed that patients in the OLZ group had a significantly greater reduction in YMRS total score compared to those in the placebo group. Moreover, a significantly higher proportion of patients in the OLZ group achieved a response (≥ 50% reduction in YMRS total score) compared to the placebo group. The OLZ group also had a significantly higher proportion of patients achieving remission (YMRS total score ≤ 12) compared to the placebo group. The trial also evaluated secondary endpoints, including changes in the CGI-BP-S score and adverse events. The results showed that patients in the OLZ group had a significantly greater reduction in the CGI-BP-S score compared to the placebo group. Adverse events reported were generally mild to moderate in severity, with the most common being somnolence, dry mouth, and weight gain [66].

In essence, OLZ/SAM was approved by the FDA for the treatment of schizophrenia and bipolar I disorder in adults, and it is important because it provides a new treatment option that may reduce the risk of weight gain commonly associated with OLZ monotherapy. The findings are summarized in Table 3.

**Table 3:** Medications approved by FDA in 2021 NDRI: norepinephrine dopamine reuptake inhibitor; SNRI: serotonin-norepinephrine reuptake inhibitor; D2: dopamine type 2; 5-HT2: serotonin type 2

Approval date	Brand name	Active ingredient	Mechanism of action	Indications	Adverse effects
3/2/2021	Azstarys	Serdexmethylphenidate and Dexmethylphenidate	CNS Stimulant NDRI	Attention Deficit Hyperactivity Disorder	Decreased Appetite, Insomnia, Headache, Upper Abdominal Pain, Upper Respiratory Tract Infection, Mood Swings, Irritability, Cough, Vomiting, Dry Mouth, Rhinorrhea
February 4, 2021	Qelbree	Viloxazine	SNRI	Attention Deficit Hyperactivity Disorder	Tachycardia (28%), Increased Diastolic Blood Pressure [19%], Drowsiness (12%), Headache (12%)
May 28, 2021	Lybalvi	Olanzapine and samidorphan	D2 receptor antagonist 5-HT2 receptor antagonist μ-opioid receptor antagonist	Schizophrenia (adults) Bipolar I Disorder (adults)	Weight Gain (19%), Increased Prolactin (males: 33%, females:41%), Increasd Triglycerides (14%)

In the year 2022

Daridorexant For Insomnia

Daridorexant is a dual orexin receptor antagonist that was approved by FDA in January 2022. It uses the active ingredient daridorexant to treat adult patients with insomnia. Daridorexant offers an alternative to traditional hypnotic medications such as benzodiazepines and nonbenzodiazepines [67]. The orexin (hypocretin) system is composed of two types of orexin receptors: OX1, which is primarily involved in regulating wakefulness and maintaining arousal, and OX2, which is involved in regulating the sleep-wake cycle and promoting wakefulness [68]. Daridorexant specifically targets both OX1 and OX2 orexin receptors, similar to lemborexant, but it may have a stronger affinity for the OX2 receptor. Thus, daridorexant may be better able to reduce wakefulness and promote drowsiness compared to other dual orexin receptor antagonists with a weaker affinity for the OX2 receptor, leading to improved sleep onset and maintenance [69]. However, daridorexant is contraindicated in patients with narcolepsy [70].

The FDA approval of daridorexant was based on the results of three randomized, double-blind, placebo-controlled Phase-III clinical trials, which involved a total of over 1,300 participants with insomnia. In these studies, participants were randomly assigned to receive either daridorexant or placebo once daily at bedtime for four weeks. The primary endpoint of these studies was the change in subjective total sleep time (sTST) from baseline to week 4, as measured by a sleep diary. Secondary endpoints included other measures of sleep quality and duration, including objective total sleep time (oTST), WASO, and LPS. The results of these studies showed that daridorexant significantly improved subjective and objective measures of sleep duration and quality compared to placebo (p < 0.05). Specifically, participants who received daridorexant had a greater increase in sTST, oTST, and LPS, and a greater decrease in WASO compared to those who received placebo. In addition, daridorexant was generally well-tolerated, with the most common adverse effects being drowsiness, fatigue, and headache [68,70,71].

Overall, daridorexant is a newly-approved medication for the treatment of insomnia in adults. Daridorexant has a stronger affinity for the OX2 receptor compared to other dual orexin receptor antagonists. By blocking the action of these neurotransmitters, daridorexant helps to promote drowsiness and improve sleep quality [72]. Daridorexant is also associated with a relatively low risk of dependence and withdrawal symptoms, making it an attractive option for those with chronic insomnia. However, like other sleep medications, daridorexant can cause drowsiness and impair one's ability to operate a vehicle or machinery. Patients who take daridorexant may also experience adverse effects such as headache, dizziness, and fatigue. It is important to follow the recommended dosage and to avoid consuming alcohol or other sedatives while taking daridorexant.

DXM/BUP For MDD

The combination of DXM/BUP was approved by FDA in mid-August 2022 for the treatment of MDD in adults [73]. DXM is a non-competitive NMDA receptor antagonist that simultaneously acts as a sigma-1 receptor agonist and inhibitor of serotonin and noradrenaline transport proteins. The antagonism of the NMDA receptor modulates glutamate neurotransmission while the agonism of the sigma-1 receptor modifies glutamate and monoamine signaling to produce antidepressant effects. On the other hand, BUP inhibits cytochrome P450 2D6-mediated DXM metabolism to increase its plasma levels and prolong its half-life, thereby enhancing its antidepressant effects [73].

Due to the lack of direct evidence for the hypothesis that depression arises due to a deficiency in monoamine neurotransmitters, the focus shifted to the downstream effects of the neurotransmitters, such as the role of glutamatergic signaling in the maintenance of neuroplasticity. The repurposed use of DXM/BUP stemmed from the growing body of research on the use of ketamine and esketamine for MDD [74]. Ketamine has been used as a fast-acting anesthetic in clinical practice for several decades and has recently gained interest as a potential treatment for MDD due to its rapid antidepressant effects by acting on the glutamatergic system. Unfortunately, ketamine and esketamine possess many unwanted adverse effects, such as hemodynamic instability, emergence reactions, respiratory depression, pediatric neurotoxicity, and drug-induced liver injury, that impact their routine use in daily practice. Hence, DXM/BUP was developed by combining the ketamine-like DXM with the well-established antidepressant BUP to produce a synergistic therapeutic effect without inducing dissociative effects [73]. The efficacy and safety of DXM/BUP for the treatment of MDD in adults was determined via a myriad of clinical trials, three of which are discussed below.

The GEMINI clinical trial was a Phase-III randomized, double-blind, placebo-controlled, multicenter study conducted to evaluate the safety and efficacy of DXM/BUP in patients with MDD. The trial enrolled 327 patients with MDD, aged 18-65 years, and randomly assigned them to receive either DXM/BUP or placebo. The primary endpoint of the study was the change in the MADRS total score from baseline to week 6. The results of the study showed that DXM/BUP was associated with a statistically significant improvement in depressive symptoms compared to placebo as indicated by the difference between the two groups: patients in the DXM/BUP group had a mean reduction of 16.6 points in the MADRS total score, while patients in the placebo group had a mean reduction of 11.9 points. DXM/BUP was also well-tolerated. The most common adverse events reported in the DXM/BUP group were dizziness, nausea, headache, diarrhea, somnolence, and dry mouth [75].

Axsome Therapeutics conducted two clinical trials, the Phase-III COMET Long-Term Trial and the COMET-AU Trial, to evaluate the safety and efficacy of DXM/BUP in patients with MDD. COMET was a long-term, open label study that enrolled 876 patients with MDD who had completed earlier DXM/BUP studies or were newly enrolled patients. The trial evaluated the safety and efficacy of DXM/BUP over a treatment period of up to one year. COMET-AU was a sub-study of the COMET trial that evaluated 115 patients with antidepressant unresponsive (AU) MDD, defined as patients with ongoing symptoms of depression despite previously receiving one standard antidepressant pharmacotherapy. The COMET study and COMET-AU sub-study took place in the United States. The trial evaluated the safety and efficacy of DXM/BUP over a six-week treatment period [76].

The results from COMET and COMET-AU showed that DXM/BUP was associated with improvements in depressive symptoms. In the COMET trial, newly treated patients had a mean reduction of 23.0 points in the MADRS total score from baseline to week 52. In the COMET-AU sub-study, treatment with DXM/BUP was associated with a mean reduction of 19.1 points in the MADRS total score from baseline to week 6. DXM/BUP was also generally well-tolerated in the COMET and COMET-AU. The most common adverse events in the COMET trial were dizziness, nausea, headache, dry mouth, and decreased appetite [76].

The EVOLVE trial was an open-label, long-term study that evaluated the safety and efficacy of Auvelity in 186 patients with MDD who had not responded adequately to one or more prior antidepressants. The primary endpoint of the study was the change in the MADRS total score from baseline to week 6. Secondary endpoints included response rates, remission rates, and changes in anxiety symptoms. The results of the study showed that DXM/BUP was associated with a statistically significant improvement in depressive symptoms from baseline. Patients had a mean reduction of 20.4 points in the MADRS total score from baseline to Week 6. DXM/BUP was also associated with significant improvements in anxiety symptoms from baseline to Weeks 1, 2, and 6. Additionally, remission of depression (MADRS ≤10) was achieved by 16%, 32%, and 46% of patients at weeks 2, 4, and 6, respectively. DXM/BUP was generally well-tolerated, with a safety profile similar to that observed in previously reported trials [77].

In essence, DXM/BUP is a rapid-acting medication that achieves pharmacological synergy and produces a robust decrease in depression scale ratings compared to BUP alone [78,79]. The findings are summarized in Table 4.

**Table 4:** Medications approved by FDA in 2022 NMDA: N-methyl-D-aspartate; NDRI: norepinephrine dopamine reuptake inhibitor

Approval date	Brand name	Active ingredient	Mechanism of action	Indication	Adverse effects
January 7, 2022	Quviviq	Daridorexant	Dual orexin receptor antagonist	Insomnia	Drowsiness (6%), Impaired Ability to Operate a Vehicle, Headache (6%), Fatigue (6%)
August 23, 2022	Auvelity	Dextromethorphan and bupropion	NMDA receptor antagonist NDRI	Major Depressive Disorder (adults)	Dizziness (15%), Nausea (13%), Diarrhea (7%), Xerostomia (6%)

Discussion

These new psychiatric medications with novel mechanisms of action with improved efficacy and safety are expected to provide further options for treating mental health disorders; promising results will lead to new patterns of research in years to come. Furthermore, the trends of FDA approvals can tell us the niche in psychiatric medicine that each medication attempts to fill.

For example, ADHD first-line medications involve CNS stimulants, which is emphasized by the FDA approval of SDX/d-MPH. SDX/d-MPH includes the commonly prescribed d-MPH but works to prolong the effective dose with the addition of its prodrug SDX. This allows both a rapid onset and prolonged duration of action. On the other hand, viloxazine acts as a NDRI with non-stimulatory effects. This signifies an attempt to create a treatment for ADHD to mitigate abuse liability and addiction from excessive CNS stimulation in patients who experience adverse events to first-line medications, such as fast heart rate, trouble sleeping, or decreased appetite. Interestingly, both of these medications were approved in 2021 implicating that ADHD treatment continues to be a subject of high interest in the field of psychiatry.

For the treatment of EDS and narcolepsy, we analyzed two medications: pitolisant and solriamfetol. There is overlap in the treatment of ADHD and narcolepsy, as CNS stimulants are first-line medications for each. Solriamfetol uses a similar mechanism of action to the first-line medications of narcolepsy by acting as a norepinephrine and dopamine reuptake inhibitor; however, it attempts to reduce the side effect profile for patients who cannot tolerate the adverse effects of amphetamines. Pitolisant is a first-in-class alternative, acting as a histamine 3 antagonist and inverse agonist, which is a novel mechanism of action for the treatment of EDS and narcolepsy. Pitolisant is also the first FDA approval for narcolepsy that is not considered a controlled substance, decreasing the risk for abuse and addiction and barriers to access. Both medications were FDA-approved in 2019, signifying interest in sleep disorders as well. Both solriamfetol and pitolisant offer alternatives in the treatment of narcolepsy by limiting the adverse effects associated with CNS stimulants.

Lemborexant and daridorexant are both orexin antagonists and two FDA approvals that capitalized on the discovery of the role of orexins in wakefulness. Current first-line therapies for insomnia include improved sleep hygiene and cognitive behavioral therapy; however, BZDs or non-BZDs are usually indicated for the treatment of insomnia with medication [80]. Orexin antagonists such as lemborexant and daridorexant are neither BZD nor are they BZD-like, indicating an attempt to integrate medications that do not carry the side effect profile of BZDs. Although BZDs and orexin antagonists are both considered class IV controlled substances, further research on orexin antagonist therapy will lead toward more medications on the like, providing a more targeted approach for the treatment of insomnia.

Lumateperone and OLZ/SAM are both atypical antipsychotics that are used for the treatment of schizophrenia. They can also be combined with mood stabilizers for the treatment of bipolar 1 disorder. OLZ/SAM attempts to reduce adverse effects of OLZ such as weight gain with the use of SAM. OLZ/SAM does offer weight reduction; however, it has not demonstrated a significant change in metabolic parameters, including insulin, glucose, hemoglobin A1c, triglycerides, and other lipids [81]. Current medication developments seem to be focused on reducing side effects and increasing efficacy rather than finding different mechanisms of action to treat schizophrenia.

The atypical antidepressant BUP along with DXM has been indicated for the treatment of MDD. Interestingly, DXM is used as a cough suppressant but acts on NMDA, glutamate-1, and sigma-1 receptors, which are all implicated in the pathophysiology of depression. BUP inhibits CYP2D6 which potentiates the effects of DXM by inhibiting its metabolism. The FDA approval of Auvelity along with the current use of ketamine and esketamine for depression show that research is moving towards the relevance of the NMDA receptor and glutamate as a treatment target for MDD. This could be helpful to adults with MDD who insufficiently respond to monoamine-based antidepressants.

Brexanolone was the first medication to be approved for the treatment of PPD, suggesting that there is a potential to target medications for depression based on specific neural pathways. Brexanolone, which is a neurosteroid made from progesterone, could be a foundation to inspire more research concerning PPD.

Lofexidine uses a different mechanism of action for the treatment of opioid withdrawal based on its effect as an alpha 2 agonist. It offers a non-opioid alternative to the treatment of withdrawal symptoms without a significant impact on blood pressure (relative to other alpha 2 agonists) [12]. Although it is considered a different mechanism of action for opioid withdrawal treatment, lofexidine was a preexisting medication that was used to treat hypertension. However, its FDA approval may also lead to further development of non-opioid medication to aid in substance use disorder treatment. 

Bremelanotide also uses a different mechanism of action to treat HSDD in women by activating melanocortin receptors. It is only the second drug after flibanserin to treat HSDD leaving great potential for further developments in this area.

Study limitations

Our research aimed to analyze and summarize characteristics of FDA-approved psychiatric medications; however, these do not include current use of psychiatric medications that have not been approved by FDA to treat a symptom or disorder (i.e. off-label utilization). Since the practice of off-label utilization is common in the field of psychiatry, this highlights a divide between the clinical experience of healthcare providers and current advancements in psychiatric research. Further research could be focused on this matter to give a more inclusive review of medications that are prescribed in practice.

## Conclusions

In this review, we looked at the psychiatric medications approved by the FDA during 2018-2022, established the clinical need for each, and discussed future directions in drug development. Although there are more “first-in-class” FDA-approved medications for psychiatry in recent years, development still falls short compared to other fields of medicine. Also, the proposed use of different mechanisms of action and new indications to treat a disorder suggest there is still a long way to go for individualized psychiatric treatment and reduced side effects. Along with the growing population and incidence of mental health disorders, the additional funding, interest, and impact of new research on psychiatric treatments will need to continually be addressed to reduce the disease burden and help patients in need.
