# Correction: A Comprehensive Review of Novel FDA-Approved Psychiatric Medications (2018-2022)

**DOI:** 10.7759/cureus.c207

**Published:** 2025-01-21

**Authors:** Shannon Giliberto, Rhea Shishodia, Meredith Nastruz, Chamandeep Brar, Sadeepa Bulathsinhala, Jonathan Terry, Sudhakar Pemminati, Sudhakar K Shenoy

**Affiliations:** 1 Department of Biomedical Education, California Health Sciences University College of Osteopathic Medicine, Clovis, USA; 2 Department of Biomedical Education, St. George's University School of Medicine, True Blue, GRD; 3 Department of Specialty Medicine, California Health Sciences University College of Osteopathic Medicine, Clovis, USA; 4 Department of Psychiatry, Clarity Clinic, Chicago, USA

This article has been corrected to fix several inaccuracies in the cited data. As Auvelity was a brand-new FDA approved medication in 2023, there was limited information concerning the new medication at the time of the initial submission. Some resources included conflicting numbers. This data has now been corrected to ensure accuracy. The authors regret that these errors were not identified prior to publication.

